# Influence of treatments in the quality of Nile tilapia (*Oreochromis niloticus*) fillets

**DOI:** 10.1002/fsn3.33

**Published:** 2013-04-26

**Authors:** Gustavo Graciano Fonseca, Angela Dulce Cavenaghi‐Altemio, Mariangela de Fátima Silva, Verônica Arcanjo, Eliana Janet Sanjinez‐Argandoña

**Affiliations:** ^1^ Laboratory of Bioengineering Faculty of Engineering Federal University of Grande Dourados Dourados Mato Grosso do Sul Brazil; ^2^ Laboratory of Food Technology Faculty of Engineering Federal University of Grande Dourados Dourados Mato Grosso do Sul Brazil

**Keywords:** Chlorine, fish fillet, microbiology, Nile tilapia, quality, shelf life

## Abstract

In this study, the influence of different treatments was evaluated: nonwashing, washing in chlorinated water and washing/soaking in chlorinated water and sodium chloride on the quality of Nile tilapia (*Oreochromis niloticus*) fillets during storage at 1.0 ± 0.5°C through daily analysis of pH, moisture, and lipids content. Microbiological analysis and growth modeling for mesophilic and psychrotrophic bacteria and *Staphylococcus* sp. were also carried out. Finally, we confirmed the microbiological safety through sensory analyses. The main results suggest that fillets washed or soaked in chlorinated water and sodium chloride present clear and narrower blood line coloration; that is, they are less oxidized than those that received other treatments and are microbiologically safe for use within 12 days of storage. It was concluded that the treatments in chlorinated and salinized water favored the quality maintenance of fillets.

## Introduction

The intensive farming of tilapia, *Oreochromis* sp., is rapidly expanding and is the second most widely farmed fish in the world, after carps. China produces almost half of the worlds' tilapia, usually commercialized as frozen products (FAO 2005). Several species of tilapia are cultured commercially, but Nile tilapia *Oreochromis niloticus* is the predominant cultured species worldwide. It presents rusticity, good growth rate, and adaptability to confinement, producing a tasty white‐color meat (Oliveira Filho et al. 2010). Nile tilapia was introduced to developing countries and cultured at a subsistence level to meet local protein needs. With the improvement of production techniques, tilapia moved into the mainstream seafood markets, and then to exportation. Tilapia exportation initially consisted of frozen whole fish, but the U.S.A. market prefers fresh fillets (FAO 2005‐2013).

Although aquaculture products are as safe and wholesome as wild‐caught species, there are some public health hazards associated with ignorance, abuse, and neglect in aquaculture technology (Garrett et al. 1997). Various aspects of microbial flora associated with fish include the changes in flora during storage, the effects of handling on microbial flora which might lead to deteriorations, and the relationship between environmental and fish microbial flora (Cahill 1990). For example, if the aquaculture production system is intensive and depends on the addition of agricultural by‐products to provide feed for the fish being raised, it will influence the microbiology of the product harvested from the ponds (Feliciano 2009).

Seafoods are highly perishable due to their autolytic enzymes and postmortem changes in pH that favor microbial growth. The handling practices since capture until storage influence the shelf life (SL) of these products. Among the most important meat quality attributes for consumers are the color, its ability to retain water as well as its tenderness and juiciness. The color is due to presence of pigments which may change color in function of chemical and biochemical reactions during processing and storage, thus being a quality indicator (Huss 1988; Ando et al. 2005; Kristinsson et al. 2005; Cyprian et al. 2013). Many factories have been searching for a way to solve the problem of color retention for tilapias or other red muscle treatment, but there has been a lack of information on how euthanasia affects fish quality, especially red muscle color (Li et al. 2008).

The use of sanitizing agents contributes to increasing the SL of chilled product. The use of chlorine and ozone are the most common. According to Lempek et al. (2001), washing with brine aims at removing the excess of blood in the meat and washing with chlorinated solutions helps inhibiting the proliferation of microorganisms on the surface. There are still many questions about the use of chlorine or not, however, since 2008, after different sessions addressed to the issue, the *Codex Alimentarius* Commission published in the document CX/CF 09/3/3 Rev. an executive summary with conclusions and recommendations favorable to the use of active chlorine in food processing (FAO 2009). The aim of this study was to evaluate the influence of treatments: nonwashing, washing in chlorinated water, and washing/soaking in chlorinated water and sodium chloride on the quality of Nile tilapia fillets.

## Material and Methods

### Sampling

Fresh Nile tilapias were kindly supplied by a local fish processing industry. Fillets were removed from Nile tilapia fish, washed, and immediately stored under refrigeration at 1.0 ± 0.5°C for 15 days. Samples were divided into three groups according to treatment received in the industry: nonwashed samples (A), samples washed with 5 ppm chlorinated water spray (B), and samples washed with 5 ppm chlorinated water spray and soaked in 20 ppm chlorinated water with 1% NaCl (C).

### Physical and chemical analysis

pH, moisture, and lipid content analyses were carried out according to AOAC (2000). Color was determined in a spectrophotometer with Barrelino^®^ accessory, employing the CIELab color system (L, a*, and b* parameters). Water retention and/or exudation was determined by weigh difference of the commercial boxes. The set of fillets of each box was weighted before washing (A), after washing (B), and after washing/soaking (C). Boxes with fillets that received the (C) treatment were stored at 1.0 ± 0.5°C for 12 days and weighed on days 2, 4, 7, 10, and 12.

### Microbiological analysis

Microbiological analyses of the fillets were performed daily for each of the three groups of samples. A representative product sample of 25 g was transferred to a Stomacher‐bag and homogenized for 60 sec in a stomacher with 225 g chilled saline peptone diluent (0.85% NaCl with 0.1% peptone). Further appropriate 10‐fold dilution of the homogenate were prepared with saline peptone diluent. For each dilution blank, replicas were prepared. 0.1 mL from each appropriate dilution step was spread on the surface of dried media into Petri dishes. The counting plate analyses were followed by classical methodology. Mesophilic bacteria were determined by using plate counting agar in deep (35°C, 48 h) and psychrotrophic bacteria by using plate counting agar in surface (20°C, 120 h). *Staphylococcus* sp. were determined by using Baird‐Parker agar with sterile 1% potassium tellurite solution and sterile egg yolk emulsion (35°C, 48 h).

### Microbial growth modeling

Modified Gompertz model (eq. 1) and modified logistic model (eq. 2) were utilized to describe the bacterial growth curves of Nile tilapia fillets (Zwietering et al. 1990):

$$\ln\frac{N}{N_{0}}=A\cdot \exp\{-\exp\left[\frac{\mu_{\max}\cdot 2\cdot 718}{A}.\left(\lambda -t\right)+1\right]\}$$

$$\ln\frac{N}{N_{0}}=\frac{A}{\{1+\exp\left[\frac{4\cdot \mu_{\max}}{A}\cdot \left(\lambda -t\right)+2\right]\}}$$

where *N* is the microbial population (CFU g^−1^) at time (h); *N*_0_ is the initial microbial population (CFU g^−1^); *A* is the asymptote (ln *N*_max_/*N*_0_); *μ*_max_ is the maximum specific growth rate (h^−1^) during the exponential growth phase, defined as the tangent in the inflection point per hour; and λ is the lag time (h). The three parameters (A, *μ*_max_, and λ) were optimized by nonlinear regression.

### Sensory analysis

The sensory analysis was performed by an untrained sensory panel of at least 39 people. Odor, color, and texture were evaluated using a simple 5 point scoring system (+2, +1, 0, −1, −2), as follows: +2 = liked much more than sample R; +1 = liked more than sample R; 0 = liked the same than sample R; −1 = dislike slightly than sample R; −2 = disliked much more than sample R (reference sample from day 0). Nile tilapia fillets were stored at 1.0 ± 0.5°C during 3, 6, 9, 12, and 14 days. Samples were then cut into 2‐cm cubes, immersed in water containing 3% NaCl, and kept in this solution for 5 min. After soaking, the samples were cooked in a microwave oven and kept under heating (50°C) until sensory evaluation. Samples were served in disposable containers, coded with three digit random numbers. Analyses were carried out in 3 days. Three samples were analyzed each day to avoid sensory fatigue, and one sample was identical to the reference sample and two samples from different storage times. Overall acceptation was evaluated in terms of purchase intention (would purchase/would not purchase). The statistical analysis was performed by ANOVA using the Statistica v.8.0 software, and means were compared by the Tukey test (5% probability) using Microsoft Excel.

### Determination of counting parameters and SL

The initial counting (*X*_0_) and maximum counting (*X*_max_) were determined by the logarithm of the lowest and highest values for microbial counting, respectively. The SL of the products was determined as the time necessary for each microorganism reaches the microbial counting established as safe, combined with the results obtained by the sensory analysis (Galarz et al. 2010).

## Results and Discussion

### Physical and chemical analysis

Average initial weight of nonwashed (A) tilapia fillets was 4556 g per box, after treatment (B) the weight was 4608 g, and after treatment (C) the weight was 4627 g, which represents an increase of 1.15%. The observed water retention in Nile tilapia fillet during storage and mass loss due to exudation indicate that there was no significant water absorption in the fillets as a result of the treatments.

pH kinetics of tilapia fillets stored at 1.0 ± 0.5°C for 15 days showed that products subjected to treatments (B) and (C) presented stability in maintaining pH in 6.6 ± 0.1 during all storage, except for nonwashed products (A), which presented pH very close to the maximum acceptable for fish (pH 7.0) (Oliveira et al. 2008) after the 9th day of storage until the end of storage (data not shown). The pH increase observed can promote the bacterial growth and therefore influence the food security for human consumption. However, these values are very similar to those reported elsewhere for tilapia fillets (Souza et al. 2004; Soccol et al. 2005; Ferreira et al. 2007; Oliveira et al. 2008; Cyprian et al. 2013).

Initial moisture of the samples from treatments (A), (B), and (C) were 79.28%, 79.79%, and 79.69%, respectively. After 12 days of storage, water loss was 0.16%, 0.54%, and 0.16% for treatments (A), (B), and (C), respectively.

Lipid content present in the samples was evaluated in order to assess possible color changes in function of fatty acids oxidation during storage. The small variation (1–2%) observed in kinetics (data not shown) was attributed to the variability of fillets, once that the analysis variance showed no significant effect compared with the control sample. The slight reduction or loss of lipids could be explained by its degradation. To verify this hypothesis, a refractive index analysis was conducted. For this analysis, control, minimum, and maximum values observed during storage were 1.465, 1.464, and 1.466, respectively, which indicated that there was no lipid oxidation during the 15 days of storage. To confirm the hypothesis that 20 ppm chlorinated water with 1% NaCl (C) had favored the color retention, it would be necessary to perform a sensory evaluation, since the color perception encompasses individual's psycho‐physiological factors. However, as higher the L value, clearer is the fillet, being more attractive to panelists, which associate the fish clarity to the product freshness.

**Figure 1:**
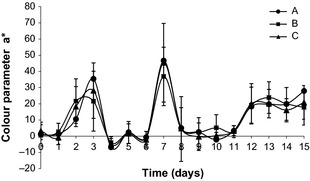
Color parameter a* (red) during storage in nonwashed (A), washed with 5 ppm chlorinated water spray (B), and washed with 5 ppm chlorinated water spray and soaked in 20 ppm chlorinated water with 1% NaCl (C) Nile tilapia fillet samples.

**Figure 2:**
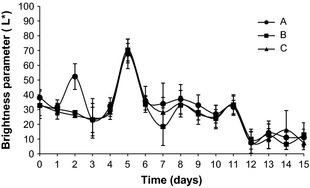
Brightness parameter l (lightness) during storage in nonwashed (A), washed with 5 ppm chlorinated water spray (B), and washed with 5 ppm chlorinated water spray and soaked in 20 ppm chlorinated water with 1% NaCl (C) Nile tilapia fillet samples.

During storage were observed changes in the parameter a* (red) as shown in Figure 1. The a* behavior was variable until 7th day, probably due to the presence of free water in the fillet, and the predominance of the L* parameter (Fig. 2) was observed. Between 8th and 11th days, there was color stabilization in all treatments, presenting values close to those of the control sample (time 0). In this period, the sample clarity slightly decreased and remained stable, being lower in washed samples. From the 12th day, a* values increased significantly (*P *<* *0.05), remaining stable until the 15th day, which shows that there was an increase in the intensity of red color. This trend was also observed for b* parameter (data not shown), influencing the reduction of L* parameter and the darkening of the fillet blood line. Samples treated with 20 ppm chlorinated water with 1% NaCl (C) had favored the color retention.

**Figure 3:**
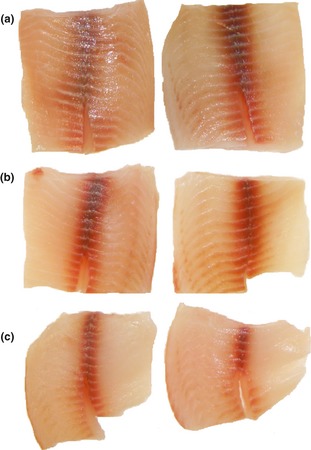
Nonwashed (a), washed with 5 ppm chlorinated water spray (b), and washed with 5 ppm chlorinated water spray and soaked in 20 ppm chlorinated water with 1% NaCl (c) Nile tilapia fillet samples after 15 days of storage.

Figure 3 shows the pictures of nonwashed (A), washed with 5 ppm chlorinated water spray (B), and washed with 5 ppm chlorinated water spray and soaked in 20 ppm chlorinated water with 1% NaCl (C) Nile tilapia fillets after 15 days of storage. It can be observed that (C) treated Nile tilapia fillets presented the blood line (red line) coloration clear and narrower, that is, less oxidized, than those that received the other treatments (A and B).

Tilapia fillets washed with 5 ppm chlorinated water spray (B) and washed with 5 ppm chlorinated water spray and soaked in 20 ppm chlorinated water with 1% NaCl (C) slowed pH rise, decreased moisture content provided better color conservation in relation to the nonwashed fillets (A).

### Microbiological analysis

Despite Enterobacteriaceae and Listeria species are recognized as the main hygiene indicator organisms, *Staphylococcus species* can produce enterotoxins and cause foodborne disease (HPA 2009). Growth kinetics of *Staphylococcus* sp. revealed that regardless of utilized treatments, the tilapia fillets showed safe for consumption during the 15 days storage (data not shown). *Staphylococcus* sp. remained stable at low levels of detection for the three treatments given to the tilapia fillets. It was observed that initial counting was approximately 1.4–1.6 log_10_ CFU g^−1^. The bacteria reached its highest value, 4.57 log_10_ CFU g^−1^, on the eighth day of storage at 1 ± 0.5°C. Low counts are usually expected in foods once their presence can be related to hygiene during processing, being man the main agent of contamination by improper handling of products (Galarz et al. 2010). It was reported that around 10^5^ and 10^6^ colony‐forming units of *Staphylococcus* sp. per gram of food can produce toxins at levels capable of causing intoxication (Lindqvist et al. 2002).

**Figure 4:**
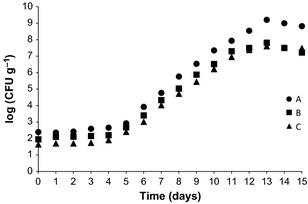
Aerobic psychrophilic microorganisms growth during storage in nonwashed (A), washed with 5 ppm chlorinated water spray (B), and washed with 5 ppm chlorinated water spray and soaked in 20 ppm chlorinated water with 1% NaCl (C) Nile tilapia fillet samples.

The characteristic change in chilled meat under aerobic conditions is a phenomenon that occurs on the surface, where sensory alterations due to the metabolites resulting from microbial growth are detected. Although the counting of aerobic psychrophilic microorganisms indicates the degree of deterioration of chilled foods, the Brazilian Legislation does not establish standard for these organisms. However, the ICMSF (1978) establishes 10^6^ to 10^7^ CFU g^−1^ as standard. This range was utilized in this study to define the microbiological SL of Nile tilapia fillets. Other authors state values ranging from 10^6^ to 10^8^ CFU g^−1^ (Davies and Board 1998). Considering an average of 10^7^ CFU g^−1^ as microbiological standard for aerobic psychrophilic microorganisms, the Nile tilapia fillets remained suitable for consumption until the 12th day (treatment A) or 14th (treatments B and C), as shown in Figure 4. The obtained values for it are in accordance with data reported for fish fillets under icing and refrigerated storage conditions (Pastoriza et al. 1994; Reddy et al. 1995).

**Figure 5:**
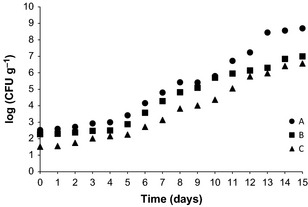
Aerobic mesophilic microorganisms growth during storage in nonwashed (A), washed with 5 ppm chlorinated water spray (B), and washed with 5 ppm chlorinated water spray and soaked in 20 ppm chlorinated water with 1% NaCl (C) Nile tilapia fillet samples.

The lower initial bacterial count was observed for fillet under treatment (C) and the highest initial contamination in nonwashed fillet (A). The fillet submitted to treatment (B) presented an intermediary contamination. This observation extended to *Staphylococcus* sp. (data not shown). For aerobic mesophilic microorganisms, this difference was not so evident, because the growth curves sometimes almost overlap, independently of fillet treatment (Fig. 5).

The obtained values during microbiological analysis showed good sanitary condition of the samples at all treatments. However, despite the small difference observed in the microbiological aspect, better results were obtained with treatments with chlorinated water. According to the microbiological safety parameter adopted (10^7^ CFU g^−1^), 14 days at 1°C proved to be the limit to ensure the quality of the (C) treated fillets (Fig. 5). This value is in the range of 2–14 days. In the present study, a relationship between the decrease in microbial load on tilapia fillets due to the increase in chlorine concentration was observed, which is in accordance with the sanitizer action reported for chlorine. There are several studies reporting the reduction of microorganisms in fish treated with chlorine. Scherer et al. (2004) observed a decrease in aerobic mesophilic and psychrotrophic microorganisms on grass carp in relation to the control group (not chlorinated). Wempe and Davidson (1992) found that the treatment of grass carp fillets with a solution of 200 ppm sodium hypochlorite significantly reduced the initial populations of coliform and total mesophilic microorganisms.

Finally, growth curves of aerobic mesophilic and psychrotrophic microorganisms, and *Staphylococcus* sp. were obtained with data from treatments (A, B, C) (data not shown), allowing calculation of parameters (*A*,* μ*_max_, and λ) for predictive applications. It was observed that maximum asymptote (A) was observed (averages) for aerobic mesophilic and psychrotrophic microorganisms for samples from treatment A (nonwashed). The maximum specific growth rate (*μ*_max_) was lower for samples from treatment C (chlorinated and salinized). However, *μ*_max_ for aerobic mesophilic and psychrotrophic microorganisms did not differ statistically (*P *>* *0.05). Lag phase (λ) slightly decreased with the treatments for *Staphylococcus* sp. (Table 1).

**Table 1:** Values of parameters *A*,* μ*_max_, and λ obtained from the modified Gombertz model for aerobic mesophilic and psychrophilic bacteria, and *Staphylococcus* sp. grown at 1°C, present on Nile tilapia fillets obtained after different treatments

Treatment		A				B				C			
Mo/parameter	M	*μ*_max_	*A*	λ	*r*	*μ*_max_	*A*	λ	*r*	*μ*_max_	*A*	λ	*r*
Aerobic mesophilic	G	0.042	7.1	111.6 (4.7 d)^a^	0.9981	0.040	5.9	109.1 (4.5 d)^a,b^	0.9962	0.038	6.4	107.8 (4.5 d)^b^	0.9986
	L	0.044	6.7	119.3 (5.0 d)^a^	0.9977	0.041	5.7	114.6 (4.8 d)^a,b^	0.9974	0.040	6.0	115.8 (4.8 d)^b^	0.9986
Aerobic psychrophilic	G	0.025	9.4	91.6 (3.8 d)^a^	0.9939	0.024	4.9	91.8 (3.8 d)^a^	0.9974	0.020	7.9	90.4 (3.8 d)^b^	0.9975
	L	0.028	7.3	106.8 (4.5 d)^a^	0.9922	0.026	4.5	100.4 (4.2 d)^a^	0.9945	0.023	5.7	103.9 (4.3 d)^b^	0.9973
*Staphylococcus* sp.	G	0.031^a^	3.0	71.2 (3.0 d)^a^	0.9927	0.026^b^	2.9	63.1 (2.6 d)^b^	0.9880	0.021^c^	3.0	26.9 (1.1 d)^a^	0.9917
	L	0.031^a^	2.9	74.3 (3.1 d)^a^	0.9967	0.027^b^	2.8	68.5 (2.9 d)^b^	0.9934	0.022^c^	2.8	34.6 (1.4 d)^a^	0.9946

Mo, microorganism; M, model; G, modified Gompertz model; L, modified logistic model; Nonwashed (A), washed with 5 ppm chlorinated water spray (B), and washed with 5 ppm chlorinated water spray and soaked in 20 ppm chlorinated water with 1% NaCl (C); *μ*_max_, maximum specific growth rate (h^−1^); *A*, asymptote; λ, lag time (h); d, days. Same letters in the same column means that there exists significant difference among average scores (*P* > 0.05) by the Tukey test.

It is known that the SL of meat has an inverse relationship with the initial contamination of the product (Galarz et al. 2010). This behavior was clearly evidenced when mathematical models were applied to the microbiological results. For both Gombertz and logistic models, a strong fit was found due the high correlation observed between experimental data and predicted values (Table 1).

### Sensory analysis

With the microbiological safety guaranteed, sensory analyses were performed with this same Nile tilapia fillets lot (treatment C). Sensory attribute scores for odor, flavor, and texture, and acceptation test scores for purchase intention are indicated by the panelists for Nile tilapia fillets stored at 0°C for 0, 3, 6, 9, 12, and 14 days. Table 2 shows the scores attributed by panelists for sensory odor, flavor, texture, and purchase intention. Test scores ranged in a mixed hedonic scale from +2 to −2, as follows: +2 = liked much more than sample R; +1 = liked more than sample R; 0 = liked the same than sample R; −1 = dislike slightly than sample R; −2 = disliked much more than sample R (reference sample from day 0).

**Table 2:** Sensory attribute scores for odor, flavor, and texture, and acceptation test scores for purchase intention are indicated by the panelists for Nile tilapia fillets stored at 1.0 ± 0.5°C for 0, 3, 6, 9, 12, and 14 days

Storage time (days)	Odor	Flavor	Texture	Purchase intention
0	0.0 ± 0.015	0.5^a^ ± 0.017	0.4 ± 0.014	0.20^a^ ± 0.011
3	0.2 ± 0.015	0.6^a^ ± 0.016	0.2 ± 0.015	0.06^a^ ± 0.006
6	−0.2 ± 0.017	−0.6^b^ ± 0.022	0.1 ± 0.018	0.12^a^ ± 0.011
9	0.1 ± 0.017	0.1^a^ ± 0.020	−0.3 ± 0.019	0.06^a^ ± 0.006
12	−0.2 ± 0.019	−0.4^b^ ± 0.018	−0.1 ± 0.020	0.09^a^ ± 0.008
14	−0.5 ± 0.017	−0.3^b^ ± 0.021	−0.1 ± 0.017	0.73^b^ ± 0.020

Same letters in the same column means that there exists significant difference among average scores (*P *>* *0.05) by the Tukey test.

The statistical analysis of the results showed that samples did not differ (*P *<* *0.05) in relation to the odor and texture attributes (Table 2), being considered similar to the reference sample (time 0) due the average values observed (range −0.2 to 0.4) being close to 0 (liked the same than sample R), while for flavor attribute, there was no significant difference (*P *<* *0.05) between samples stored for 3 and 9 days, being considered equal to the reference sample. For samples stored for 6, 12, and 14 days, the panel assigned scores with negative values suggesting “dislike slightly than sample R”.

In relation to purchase intention, the variance analysis of the results indicated that the fillets stored for 0, 3, 6, 9, and 12 did not differ (*P *<* *0.05). So if the product was on sale, all would be purchased. Samples with 14 days of storage presented significant differences (*P *>* *0.05) in relation to purchase intention, indicating that most of the panelists would not purchase the product. Compared to flavor results, it observes that it is in agreement with the acceptation of the potential consumer. Another feature observed is the rejection of the product after the 12‐day shelf‐life expiration (14 days) (Table 2).

**Figure 6:**
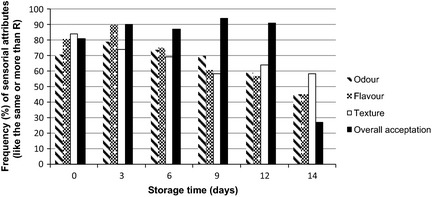
Sum of the frequencies (%) of the scores representing liked the same or more than R for the sensory attributes odor, flavor, and texture, and the overall acceptation (would purchase) of Nile tilapia fillets washed with 5 ppm chlorinated water spray and soaked in 20 ppm chlorinated water with 1% NaCl (treatment C), stored at 1.0 ± 0.5°C for 0, 3, 6, 9, 12, and 14 days.

In order to improve the perception of the sensory results, the positive values of the assigned scores were grouped in “liked the same or more than sample R”. The result of grouping is presented as the sum of the frequencies for the sensory attributes odor, flavor, and texture, and the overall acceptation (would purchase) of Nile tilapia fillets washed with 5 ppm chlorinated water spray and soaked in 20 ppm chlorinated water with 1% NaCl (treatment C), stored at 1.0 ± 0.5°C for 0, 3, 6, 9, 12, and 14 days (Fig. 6).

It observes a decreasing acceptability of the product with the storage time in relation to the odor, flavor, and texture. After 14 days of storage, odor and flavor acceptability were of 45%, suggesting rejection of the product with consequent loss of market share. However, for texture the acceptability at the 14th day of storage was of 58%. From the foregoing, it is recommended the consumption of the product until the 12th day of storage (Fig. 6).

Overall acceptation in terms of purchase intention (would purchase) is also shown in Figure 6. Samples with 14 days of storage had a major role in rejection mainly due color change of the fish blood line, also present in C treated fillets which confirms the importance of the product appearance at the time of purchase (Cyprian et al. 2013).

The three sensory attributes evaluated as well the overall acceptation in terms of purchase intention were influenced by the fillet storage time. Despite the sensory evaluation being carried out by the untrained panel, sensory perception was distinct with time, independently of the samples having been served in sequence of storage time or not. The randomness of samples presentation promoted the reliability of the obtained results which leads to conclude that the samples treated with 5 ppm chlorinated water spray and soaked in 10 ppm chlorinated water with 1% NaCl (treatment C) provided acceptable products within 12 days of storage. This result is promising, considering that was reported elsewhere that Nile tilapia fillets presented a SL of 13–15 days for air‐packaged cooked fillets samples during storage at 1°C (Cyprian et al. 2013).

## Conclusions

Nonwashed fillets of Nile tilapia provided acceptable products within 12 days of storage. Fillets washed with 5 ppm chlorinated water spray or with 5 ppm chlorinated water spray and soaked in 20 ppm chlorinated water with 1% NaCl presented microbiologically safe during 14 days. However, sensory analysis of chlorinated and salted fillets showed that the product was recommended for consumption until the 12th day of storage. It was concluded that the treatments in chlorinated and salinized water favored the overall quality maintenance of fillets.

## Conflict of Interest

None declared.
